# Complements or substitutes? Associations between volumes of care provided in the community and hospitals

**DOI:** 10.1007/s10198-021-01329-6

**Published:** 2021-06-17

**Authors:** Yiu-Shing Lau, Gintare Malisauskaite, Nadia Brookes, Shereen Hussein, Matt Sutton

**Affiliations:** 1 Health Organisation, Policy and Economics, University of Manchester, Manchester, UK; 2 Personal Social Services Research Unit, University of Kent, Kent, UK; 3 Centre for Health Services Studies, University of Kent, Kent, UK; 4 Department of Health Services Research and Policy, London School of Hygiene & Tropical Medicine, London, UK; 5 Melbourne Institute: Applied Economic and Social Research, University of Melbourne, Melbourne, Australia

**Keywords:** Community care, Primary care, Secondary care, Net unit costs, I10

## Abstract

Policymakers often suggest that expansion of care in community settings may ease increasing pressures on hospital services. Substitution may lower overall health system costs, but complementarity due to previously unidentified needs might raise them. We used new national data on community and primary medical care services in England to undertake system-level analyses of whether activity in the community acts as a complement or a substitute for activity provided in hospitals. We used two-way fixed effects regression to relate monthly counts of community care and primary medical care contacts to emergency department attendances, outpatient visits and admissions for 242 hospitals between November 2017 and September 2019. We then used national unit costs to estimate the effects of increasing community activity on overall system expenditure. The findings show community care contacts to be weak substitutes with all types of hospital activity and primary care contacts are weak substitutes for emergency hospital attendances and admissions. Our estimates ranged from 28 [95% CI 21, 45] to 517 [95% CI 291, 7265] community care contacts and from 34 [95% CI 17, 1283] to 1655 [95% CI − 1995, 70,145] GP appointments to reduce one hospital service visit. Primary care and planned hospital services are complements. Increases in community services and primary care activity are both associated with increased overall system expenditure of £34 [95% CI £156, £54] per visit for community care and £41 [95% CI £78, £74] per appointment in general practice. Expansion of community-based services may not generate reductions in hospital activity and expenditure.

**Supplementary Information:**

The online version contains supplementary material available at 10.1007/s10198-021-01329-6.

## Introduction

In many countries, substantial proportions of health care services are provided in the community rather than at hospitals. These services may be the first point of contact for individuals to the healthcare system and include a wide variety of roles such as “primary care physicians,…, nurses, pharmacists, auxiliaries and community health workers” [1]. Policymakers often assume that primary care strengthening and the improvement and expansion of care in community settings will ease increasing pressures on hospital services. For example, the recent national strategy for the health service in England described a reorganisation of how care services were to be delivered by introducing new care models [2]. These were intended to increase the integration between health care providers, ultimately providing better quality care for patients and moving away from specialist hospital care by providing more care in community settings.

It is believed that with the current ageing demographics and increased disease burden, the reliance on hospital care will be financially unsustainable in the long term [3] because it is more costly for individuals to be treated in hospitals than in the community. Therefore, the belief that health care services delivered in the community can substitute for hospital services is attractive to the health system, as lowering hospital service use is expected to reduce overall health care costs.

The overall relationship between community and hospital activity is complex and will depend on the health condition and the type of care required. There is a straightforward complementary relationship between first primary care physician (GP) appointments and elective hospital care in healthcare systems where GPs act as gatekeepers for non-emergency hospital services. There is also a straightforward substitution relationship where community and hospital services offer the same intervention and patients can choose between them based on the differences in availability. This is likely to apply, for example, where patients need urgent advice or minor treatments for relatively simple acute health problems.

There are some health conditions requiring specialised equipment and care by specialists that will require treatment in hospitals. However, more generally, some community service activities could be either complements or substitutes for non-emergency hospital services if they help manage conditions outside of the hospital setting. Such activities would include the management of wound care or patients with multi-morbidities. These activities could avoid patients experiencing worsening health conditions and therefore mitigate needs for non-emergency hospital services. However, improvements in community-based care may have complementary relationships with hospital services by uncovering previously unidentified needs. This would mean that increases in the provision of services in the community may lead to increases in hospital service use. These system-level relationships are critical to the economic case for expanding services in the community and understanding the impact on population health and wellbeing.

The mechanisms by which community-based services influence hospital services will differ between planned and unplanned reasons for care requirements. The previous literature on the relationship between primary care and planned secondary care provides mixed results. A systematic review that studied the substitution of outpatient hospital care with specialist care in a primary care setting found that most (11 out of 14) studies report a substitution effect between specialist care within a primary care setting and planned outpatient hospital care [4]. We found studies outside of the scope of this systematic review that also provide mixed findings. One study from the United States of America (USA) found a substitute effect between primary care and speciality medical encounters [5]. Another USA study found no effect of increasing primary care fees, which should reduce use, on hospital admissions and outpatient visits [6]. A study from the Netherlands found no effect of expanding services performed in primary care on GP referral behaviour [7]. A study from Norway found a weak substitution effect between long-term primary care and hospital use among older patients. Still, the reduction did not significantly relieve pressures on the hospital system [8]. Another Norway study found a complementary relationship between GP consultations and outpatient visits rates [9]. These conflicting findings from the literature may be driven by the differences in the identification and type of both primary and secondary care and differences in the institutional settings for each study.

Several studies have examined elements of these relationships between hospital- and community-based care. We summarise their main findings below. However, we are not aware of studies that have examined the effects at system level, particularly the relationships between volumes of contacts with community health care workers and the use of hospital services in any country.

This paper aims to estimate the overall effect of changes in the provision of services in community settings on hospital care in England. The availability of two new datasets on care provided in community settings within England enables us to assess empirically whether community activity acts as a complement or a substitute for hospital services. We use these newly-available national datasets to determine the system-level association between the volume of services delivered in the community (including community health services and appointments in General Practice) and volumes of hospital service activity in England. We then use the data on unit costs to estimate the effect of increasing community activity on total system expenditure.

## Methods

Before describing the datasets and how we combined and analysed them, we describe the basic institutional arrangements for health services in England and review previous studies of elements of the relationship between hospital- and community-based care.

### Institutional background

Publicly funded health care through the National Health Service (NHS) in England is generally free at the point of use and funded through general taxation. Health care services are rationed based on the health needs.

NHS England allocates a proportion of its health care services budgets to one of 135 (as of March 2021) Clinical Commissioning Groups (CCGs) who negotiate contracts for NHS health care services at a smaller regional level This allocation is based on the health care needs of the population. The population's health care needs will differ by region due to different population characteristics such as average age, deprivation and rurality.

CCGs negotiate contracts with NHS Trusts to provide hospital and or community services and General Practices for primary care services. Public Health England allocates public health budgets through 152 Local Authorities in England. These Local Authorities negotiate contracts with service providers for public health services, including some community services.

For planned care, England's health care system is structured around a gatekeeper system in which people register with a family doctor, called General Practitioner. General Practitioners are trained to identify and meet most health care needs and are responsible for, but not limited to, the management of long-term health conditions and referring patients to other health care services in line with their care needs. General Practitioners are usually the first point of contact for patients, where the care they provide is under the umbrella term of primary care. Access to specialist care delivered by hospital specialists, secondary care, is generally controlled by General Practitioners. If a General Practitioner determines that a patient will benefit from receiving specialist care delivered in a hospital, a patient can be referred for an outpatient consultation. First, outpatient consultations are made with hospital specialists responsible for hospital treatments and do not necessarily result in treatments. If treatment for a patient’s health condition requires hospital admission, hospital specialists will recommend that admission.

### Definition of community services

Community care services are diverse and challenging to define as the precise nature of services differs between health systems, between regions and between population groups. A recent definition states “[t]he main types of services delivered in the community include, but are not limited to: adult community services (e.g. district nursing, intermediate care, end of life care), specialist long term condition nursing (e.g. heart failure, diabetes, cancer) planned community services (e.g. podiatry, speech and language therapy, physiotherapy) children’s 0–19 services (e.g. health visitors and school nursing) health and wellbeing services (e.g. sexual health, smoking cessation, weight management) and inpatient community services (e.g. inpatient services)” [3]*.* Broader definitions of services provided in the community also include care delivered within General Practice [10].

#### Literature review focusing on the English health care system.

For unplanned care, patients can directly access hospital care through Accident and Emergency (A&E) departments without prior access to a General Practitioner. We would expect that patients attending accident and emergency services would present with severe health conditions. However, some patients may attend accident and emergency departments due to perceived or actual lack of access to a General Practitioner. A systematic review conducted by Flores-Mateo et al. [11] found that increasing primary care supply is associated with reductions in A&E attendances. Studies by Lippi Bruni, Mammi and Ugolini [12] and Whittaker et al. [13] have examined the impact of extending access to general practices on the use of emergency hospital services. Both studies found that increases in access led to reductions in emergency hospital service use. However, more recently, Parkinson et al. [14] examined the association of avoidable A&E attendances to primary care quality in England. The authors found that increases in primary care quality (and availability) are associated with reductions in avoidable A&E attendances. In addition, one study in the USA setting found that increasing fees at primary care services was not associated with emergency hospital service use [6].

Harrison et al. [15] examined the effect of a financial incentive scheme to improve the quality of primary care in England on avoidable emergency hospital admissions. They found that avoidable hospital admissions reduced more for conditions targeted under the financial incentive scheme by more than incentivised conditions.

A systematic review study by Bickerdike et al. [16] reviewed studies on interventions linking primary care and care in the community through social prescribing. One element of the review examined the impact of the intervention on secondary care and found that two out of three studies found social prescribing reduced secondary care activity. However, the studies had weak designs or small samples. A subsequent study by Munford et al*.* [17] found reductions in secondary care utilisation (outpatient visits, ambulance call outs and A&E attendance) between individuals who participated in community assets than those who did not; these findings were not subject to regression analyses. Their follow-up [18] also found that individuals participating in community assets have lower health care costs than individuals who do not.

A systematic review by Baxter et al. [19] reviewed the literature of integrated care within and outside of the UK, where one of the three focuses was on the usage of health care resources. The authors found that five UK studies found that integrated care is associated with reductions in outpatient appointments, whereas no international studies found a relationship. The authors found the relationship between integrated care and unscheduled care, the number of hospital admissions and A&E visits- to be mixed. The majority of studies reviewed reporting either reductions in hospital services or no effects. Morciano et al. [20] examined new integrated care models in England on emergency hospital admissions using a quasi-experimental design for policy evaluation, difference-in-differences. The authors found that the new integrated care models were associated with reductions in emergency admissions, driven by individuals within residing care homes two years post-intervention.

We have identified no evidence specific to community care services, but the literature on social care services suggests community care services could be a weak substitute for hospital services. A systematic review of the association between social care and hospital care conducted by Spiers et al. [21] found no evidence on the number of social care users and A&E attendances in the UK [22] and weak substitute relationships between users of care homes and hospital admissions [23, 24] and A&E attendances in the USA [23]. However, a study by Forder [25] assessed the relationship between long term care and hospital utilisation for older patients, finding them to be substitutes, with a £1 spend on social care leading to a £0.35 reduction in hospital expenditure. More recently, Crawford, Stoye and Zaranko [26] found that reductions in social care spending for people aged 65 and above have led to an increase in A&E utilisation. There is no direct evidence on community care services, but the literature on social care services suggests community care services could be a weak substitute for hospital services.

### Data

We used three primary datasets in our analysis, covering the 23 months between November 2017 and September 2019: (a) counts of hospital provider-level activity from Hospital Episode Statistics (HES) [27]; (b) counts of community care contacts at community service provider level from the Community Services Data Set (CSDS) [28]; and (c) counts of appointments in General Practice at Clinical Commissioning Group level from the Appointments in General Practice dataset [29]. These are all available monthly. The CSDS has been published since October 2017 and the Appointments data since November 2017.

The publicly available monthly series of hospital activity from provider-level HES captures counts of all publicly-funded hospital activity; hospital admissions, unplanned hospital admissions, outpatient visits and emergency department attendances. Providers include Trusts, Foundation Trusts and independent service providers. In total, the data contains 242 different hospital service providers.

The Appointments in General Practice data series captures all scheduled appointments from General Practice that use one of four computer systems (EMIS, TPP, MICROTEST and VISION). A study in 2018 by Kontopantelis et al. [30] found that of 7526 General Practices in 2016, 7477 (99%) of practices used these computer systems. Counts of appointments in General Practice are stratified by health care professional type (GP, other practice staff and unknown), mode of appointment (telephone, face to face, video, online, home visit and unknown), attendance status (attended, not attended and unknown) and the length of time between the booking date and the appointment date.

NHS Digital reports the number of General Practices who did not report appointments data each month in each CCG and the registered population sizes of those practices. We inflate the count of appointments to account for the expected amount of un-reported activity using the registered population sizes. We used the list of 191 CCGs that existed as of April 2019. We combined the earlier data from the CCGs that merged using information regarding CCG mergers from the Organisation Data Service [31].

The Community Services Data Set is collected on all publicly funded community services in England. Providers of publicly-funded community services are legally mandated to supply this information. Providers include Foundation Trusts, Acute Trusts, Mental Health Trusts, Community Health Care Trusts, Care Trusts, social enterprises, Integrated Care Organisations, independent sector providers and local authorities [28]. A total of 124 community service providers submitted community care contacts data to the CSDS within the study period. We use data on care contacts from the CSDS.

In each dataset, we calculated a relative activity index for each provider. We divided the count of activity reported by each provider in each month by the average activity reported by that provider across all months. This normalises the activity level for each provider to one. We include the definition and the data required to generate the relative activity indexes in Appendix Table A1. The relative activity indexes are calculated by dividing activity levels in each provider month by the provider’s mean activity level across all months and all these are shown in detail in figures.

**Table 1:** Descriptive statistics

Variable	Mean	Standard deviation	Between provider standard deviation	Within provider standard deviation
Count				
A&E attendances^^	10,518	6248	5875	2189
Hospital admissions^^^	6534	5177	5150	855
Hospital admissions non-elective^^^^	3276	2339	2332	467
First outpatient visits^	8066	6667	6620	1266
Community care contacts*	47,596	33,312	32,228	9735
GP appointments**	134,956	94,046	93,215	13,886
Index				
A&E attendances^^	1.020	0.233	0.233	0.189
Hospital admissions^^^	1.000	0.122	0.026	0.120
Hospital admissions non-elective^^^^	0.991	0.295	0.092	0.280
First outpatient visits^	1.006	0.257	0.628	0.171
Community care contacts^	0.999	0.143	0.016	0.142
GP appointments^	1.000	0.077	0.003	0.077
Index CCG population	1.000	0.007	0.001	0.007
Proportion male^	0.498	0.005	0.005	0.0004
Years of age (proportions)^				
0–14	0.171	0.014	0.014	0.001
25–64	0.646	0.029	0.029	0.001
65 and over	0.182	0.036	0.036	0.001

^Calculated on 4484 provider months

^^Calculated on 3557 provider months

^^^Calculated on 4406 provider months

^^^^Calculated on 3842 provider months

*Per community care provider per month

**Per CCG per month. Means and standard deviations are calculated using different number of provider months as there are differences in the number of organisations which are providing each type of care service over time. For example, there are more hospital providers that accept first outpatient visits than A&E attendances. Values for the indices are relative deviations from the mean value of activity. For A&E attendance, this signifies that, within the analysis sample, on average hospital A&E activity levels are 2% above the mean. The mean values of indices are not exactly one due to missing information after the calculation of the indices

### Combining the datasets

Each of the three activity datasets is available at a different level of aggregation. Hospital activity is published at the hospital Trust level, including independent sector providers that provide publicly funded care. Appointments in General Practice is published at the CCG level. Community health services activity is published for each community health care provider.

We adopted the hospital Trust as the unit of analysis as we sought to explain variations in hospital activity. There is no direct mapping of primary care providers and community care providers with hospital care providers. Therefore, we used two methods to assign General Practice and community care providers activities to each hospital provider.

Micro-data are available from HES on the hospitals used by populations registered with the General Practice in each CCG. We used the most recent micro-data available (April 2017 to March 2018) to create the weights for the primary care relative activity levels. We calculated the shares of each hospital’s activity that came from patients registered with practices in each CCG and used these shares to weight the relative activity levels for each CCG using Eq. 1.

1 $$ {\text{app}}_{ht} = \mathop \sum \limits_{p} \left( {H_{hp} \times \frac{{A_{pt} }}{{\overline{A}_{p} }}} \right), $$

where ${\text{app}}_{ht}$ denotes the attributed relative index of appointments in General Practice for hospital provider *h* and month *t*, *H* denotes a historical attribution of the proportion of patients at hospital provider *h* from General Practice *p* and *A* denotes the number of appointments at a General Practice.

For example, if 75% of a hospital Trust’s patients were registered with practices belonging to a CCG whose appointments activity was elevated by 10% and the remaining 25% of patients were registered with practices belonging to a CCG which reported its average level of appointments activity, the appointments activity index for the hospital Trust would be 1.075 = (75% × 1.1) + (25% × 1.0). The indexed value of 1.075 can then be interpreted as 7.5% more than the average number of GP appointments.

There is no national micro-data to apply the same approach for community services activity levels. Owing to the diverse nature of community care services, the commissioning and geographical footprints for each care provider are unclear and overlapping. From a survey of 71 acute trusts providing community services, on average, community trusts were commissioned by over five different organisations, with Clinical Commissioning Groups (CCGs) accounting for 77%, NHS England 5% and local authorities 17% of the overall community services budget [3, 32]. A single community service provider will be responsible for providing care to a geographical area. However, some geographical locations will have one provider providing most community care activity and multiple smaller providers providing more specific community services [10]. CCGs hold, on average, 50 different contracts for community health services [32].

We created weights based on the distances between providers and their relative sizes [33]. The distance-size weight takes values between zero and one for each possible combination of community service provider and hospital service provider.

We use the following series of Eqs. (2–5) to generate a distance-size weight between each community and hospital service provider and Eq. 6 uses the distance-size weights to create a hospital-level community activity index:

2 $$ {\text{DW}}_{ph} = \frac{1}{{\left( {d_{ph} + 1} \right)^{2} }}, $$

3 $$ {\text{SW}}_{p} = \frac{{\overline{C}_{p} }}{{\mathop \sum \nolimits_{p} \overline{C}_{p} }}, $$

4 $$ {\text{GW}}_{ph} = {\text{DW}}_{ph} \times {\text{SW}}_{p} , $$

5 $$ {\text{GW}}_{ph}^{*} = \frac{{{\text{GW}}_{ph} }}{{\mathop \sum \nolimits_{p} {\text{GW}}_{ph} }} $$

6 $$ {\text{com}}_{ht} = \mathop \sum \limits_{p} \left( {\frac{{C_{pt} }}{{\overline{C}_{p} }} \times {\text{GW}}_{ph}^{*} } \right), $$

in which, ${\text{DW}}_{ph}$ is a set of distance weights based on the distance between community provider *p* and hospital provider *h* ($d_{ph}$)*.* ${\text{SW}}$ is a set of size weights calculated using the relative size of a community provider compared to all community service providers based on $C$, the average monthly volume of community care contacts provided by community provider *p*. ${\text{GW}}$ are the distance-size weights, generated by taking the product of the distance and size weights. ${\text{GW}}^{*}$ are the normalised distance-size weights that ensure the weights assigned to each hospital service provider sum to one. The index of relative community services activity attached to hospital *h* in month *t* is then the weighted average of the indices of relative community services activities for each community provider in month *t*. We present a hypothetical example of how the distance-size weights are calculated in the appendix Table A2.

Some organisations will appear in both *p* and *h* because they provide both hospital and community services. The importance of distance to the weights depends on the power of the decay function in Eq. (2). We use a distance power of two and conduct sensitivity analysis adjusting for different powers of distance. Further details on how the weights were calculated are provided in the appendix.

We used all pairwise combinations of hospitals and community services providers when calculating the weights to be used in analysing influences on the volume of emergency department attendances and non-elective hospital admission. This is because patients could use emergency departments anywhere in the country. This situation is much less likely to happen for outpatient visits and elective hospital admissions. Therefore, we calculated weights for planned care using the five community service providers closest to each hospital Trust. All provider location information was obtained through the NHS Digital Organisation Data Service [31]

Control variables, including figures on population size, age distribution and the proportion of the population that is male from monthly series of registered patients at each General Practice, were obtained from NHS Digital [34]. Data were aggregated to the CCG level. As the population size measure, a relative population size index was calculated by dividing the monthly figures for each CCG by the CCG average. Then these covariates were attached to hospital service providers using the same weights as for the indices of relative primary care activity levels.

### Estimation strategy

We set out to estimate the following linear equation:

7 $$ {\text{hosp}}_{{{\text{ht}}}} = \beta_{0} + \beta_{1} {\text{com}}_{{{\text{ht}}}} + \beta_{2} a{\text{pp}}_{{{\text{ht}}}} + \gamma X_{{{\text{ht}}}} + \tau_{t} + \varepsilon_{{{\text{ht}}}} , $$

where ${\text{hosp}}_{ht}$ denotes the index of the relative volume of activity at hospital service provider $h$ in month $t$, ${\text{com}}_{ht}$ is the index of the relative volume of community services attached to hospital *h* in month *t*, ${\text{app}}_{ht}$ is the index of the relative volume of appointments in General Practice linked to hospital *h* in month *t*, $X$ is a vector of explanatory variables (index of relative population size, proportion of the population that is male, proportion of the population aged 0–14 years, proportion of the population aged 15–64 years and proportion of the population aged 65 years and over), $\tau$ are a set of monthly time dummy variables and $\varepsilon$ is the error term with a mean of zero. Positive values of $\beta_{1}$ and $\beta_{2}$ would indicate complementary relationships between activity in the community and hospitals, whereas negative values would indicate substitute relationships. All are estimated with robust standard errors.

We allow for time-varying unobservable factors common to all hospitals and unobservable factors specific to each hospital fixed over time. However, there may be time-varying unobservable factors that vary across hospitals and may bias the estimated relationships. One such variable that was initially explored is the income deprivation of individuals in the local area. We did not include this measure because it showed almost no changes throughout the study period and was therefore not suitable for inclusion in the fixed effects estimation.

We use Oster bounds to assess the likely importance of these unobservable factors [35]. This analysis adjusts the estimated coefficients from the model accounting for the R-squared and potential direction of bias due to not including all possible confounding variables.

The adjustment is provided by following formula:

8 $$ \beta^{*} = \overset{\lower0.5em\hbox{$\smash{\scriptscriptstyle\smile}$}}{\beta } - \delta \left[ {\dot{\beta } - \overset{\lower0.5em\hbox{$\smash{\scriptscriptstyle\smile}$}}{\beta } } \right]\frac{{R_{\max } - \overset{\lower0.5em\hbox{$\smash{\scriptscriptstyle\smile}$}}{R} }}{{\overset{\lower0.5em\hbox{$\smash{\scriptscriptstyle\smile}$}}{R} - \dot{R}}}, $$

where $${\upbeta }^{*}$$ is the bias-corrected coefficient, $$\overset{\lower0.5em\hbox{$\smash{\scriptscriptstyle\smile}$}}{\beta }$$ is the coefficient from the full model, $$\dot{\beta }$$ is the coefficient from the model without covariates, $$\overset{\lower0.5em\hbox{$\smash{\scriptscriptstyle\smile}$}}{R}$$ is the *R*-squared statistic for the full model, $$\dot{R}$$ is the *R*-squared statistic from the model without covariates, $$R_{\max }$$ is the maximum obtainable R-squared statistic and $$\delta$$ represents the importance of the unexplained confounders compared to the explained confounders.

We make three assumptions when adjusting the coefficients using this method. First, we assume that the maximum obtainable R-squared is equal to 1.3 times the *R*-squared value from the full model. Second, we assume that maximum explanatory power using all unobserved confounders is equal to that with the observed confounders. Third, we assume that the direction of bias when including observed confounders and unobserved confounders is the same.

### Costing

Healthcare supplied by the English NHS is funded through general taxation. This publically funded budget will be used to fund both activities in the community and activity provided in hospitals. With finite budgets, policymakers in England intend to move more health care services to be delivered in the community, which are considered cheaper than hospital services [2]. Net unit costs for services provided in the community is determined by the relationship between services provided in the community and hospital services. We would expect that net unit cost is lower than the unit costs of the community's service if there is a substitution effect with hospital services. The direct costs of community services will be (partially) offset by the indirect cost savings from hospital activity reductions.

We estimated the net unit costs associated with each care contact provided within the community net of any expected reductions (or increases) in hospital activity using the estimated regression coefficients, mean volumes of activity and unit costs from NHS England and Improvement for hospital and community services [36] and appointments in General Practice [37]. Unit costs for hospital and community services are the aggregated costs from the National Cost Collection for 2019, formally known as NHS Reference Costs. Unit cost for an appointment in General Practice was obtained from an NHS England article published in 2019, which divided the total cost to of General Practice to the NHS by the number of General Practice appointments.

We estimated the net unit cost of activity provided in the community using the following equation.

9 $$ \Delta {\text{Net Cost}}_{i} = c_{i} + \left( {\frac{{\mathop \sum \nolimits_{j} \left( {c_{j} \times \overline{{{\text{hosp}}}}_{j} \times \hat{\beta }_{ij} } \right)}}{{\overline{{x_{i} }} }}} \right), $$

where $\Delta {\text{Net Cost}}_{i}$ denotes the net unit cost for each community service *i* (community care contact or appointment in General Practice), $\overline{x}$ denotes the mean number of contacts in the community per month, $c$ is the unit cost of activity, $\overline{hosp}_{j}$ denotes the mean hospital activity per hospital provider per month for hospital activity *j* and $\hat{\beta }$ is an estimated coefficient from Eq. (7).

### Additional analyses

We performed a set of additional analysis to test whether the associations between care provided in the community and hospital services were sensitive to different assumptions and situations. Firstly, community services delivery—and therefore, associations with hospital services—may differ in rural and urban settings. This may be due to the increased scope of community services within rural areas compared to urban areas. We stratified our sample by the size and proportion of each hospital’s treated population who lived in rural areas based on the historical hospital activity.

Secondly, we limited the sample to hospital service providers that also provide community care services. Mainly because the attribution of community activity to the hospital is more specific, but we may expect the relationship between community care and hospital services to be more robust when provided by the same care organisation.

Thirdly, we limited the analysis to include NHS Trusts and Foundation Trusts only. All hospital services’ providers that deliver publicly-funded activity are included in the data. Independent hospital service providers generally provide much lower publicly-funded hospital activity levels than NHS Trust and Foundation Trusts. As we use relative activity levels, smaller providers will have large fluctuations in the relative activity index following small actual volume changes.

Fourthly, as community care data are a relatively new dataset, there were initial data quality issues where providers did not submit data through all months included in our study period. In our core analysis, we did not include providers with missing data. As a sensitivity analysis, if a community provider did not report data in a particular month, we linearly interpolated the missing counts using the counts in the preceding and proceeding month. We did not extrapolate data before a provider first reports or after a provider last reports in the dataset.

Fifthly, we tested the effect of changing the distance decay function when attributing community services to hospital service providers. We used a linear function of distance and then changed the power on the decay function to examine whether our results were sensitive to the distance-size power choices. The effect of increasing the distance decay function meant more weight was applied to the closest community care providers to the hospital service provider. Thus, changing the decay function altered the amount of community care services attributable to each hospital service provider.

Finally, we estimate the relationship between hospital activity and community services activity taking natural logarithms of the levels of activity in hospitals and in the community. This is to mitigate the potential effects of large outliers and the positive skew in the relative activity indices.

## Results

### Descriptive statistics

Table 1 provides descriptive statistics on the measures of health care activity and the covariates. On average, emergency departments have 10,500 attendances each month. Hospitals, on average, have 6500 hospital admissions (of which 3300 are non-elective) and 8100 first outpatient visits each month. On average, community care providers have 47,600 care contacts each month. On average, a CCG has 135,000 appointments in General Practice each month.

A&E providers also have the lowest between provider standard deviations in relation to the mean of all other service providers. With the largest standard deviation to mean ratio (and between provider standard deviation to mean ratio), first outpatient providers are more varied than CCGs and hospital service providers. Appointments in General Practice series are the most stable relative to each provider's mean with the lowest within provider standard deviation to mean ratio. A&E providers and Community care providers exhibit the largest within provider variations given their respective means.

### Main regression results

Table 2 shows the regression results from linear regression models. We find that community contacts and all hospital activity are weak substitutes, since an one-unit change in community services is associated with a − 0.16 [95% CI − 0.22, − 0.10], − 0.03 [95% CI − 0.05, − 0.002], − 0.03 [95% CI − 0.05, − 0.01] and − 0.03 [95% CI − 0.05, − 0.005] unit change in A&E attendances, non-elective hospital admissions, hospital admissions and first outpatient visits respectively. Our largest effect size is for A&E attendances, where 28 [95% CI 21, 45] community care contacts are associated with a reduction of one A&E attendance.

**Table 2:** Regression results

	Index of relative A&E attendances activity	Index of relative non-elective hospital admissions activity	Index of relative hospital admissions activity	Index of relative outpatient visits activity
Coefficient [95% confidence interval]				
Index of relative community contacts activity	− 0.162 [− 0.22, − 0.10]	− 0.028 [− 0.05, − 0.002]	− 0.03 [− 0.05, − 0.01]	− 0.026 [− 0.05, − 0.005]
Index of relative GP appointments activity	− 0.375 [− 0.74, − 0.01]	− 0.025 [− 0.13, 0.08]	0.086 [− 0.01, 0.18]	0.105 [− 0.02, 0.23]
Proportion population aged 0–14	− 0.233 [− 3.14, 2.68]	0.51 [0.10, 0.92]	0.48 [0.13, 0.83]	0.492 [0.13, 0.85]
Proportion population aged 65 and over	− 0.786 [− 2.72, 1.15]	0.121 [0.01, 0.23]	0.134 [0.04, 0.23]	0.141 [0.03, 0.25]
Proportion population male	− 5.346 [− 10.83, 0.14]	0.477 [− 0.14, 1.10]	0.391 [− 0.15,0.93]	0.376 [− 0.28, 1.03]
Index of relative CCG population size	− 3.606 [− 4.85, − 2.36]	− 0.964 [− 1.47, − 0.46]	− 0.615 [− 0.97, − 0.26]	− 0.004 [− 0.48, 0.47]
Provider months	3557	3899	4408	4486
Estimated effect sizes				
Community care contacts per hospital contact	− 28 [− 45, − 21]	− 517 [− 7265, − 291]	− 240 [− 728, − 146]	− 224 [− 1180, − 118]
GP appointments per hospital contact	− 34 [− 1283, − 17]	− 1655 [− 1995, 70145]	241 [− 2719, 256]	159 [− 3816, 175]

The results from linear regression models. Models also include provider fixed effects, monthly time effects. Indexes of relative activity are calculated by dividing monthly volume by the average volume reported by the provider over all months. All regressions are estimated with robust standard errors. Estimated effect sizes are the number of contacts or appointments in the community that would change the use of hospital service by one unit. We used the coefficients and mean activity levels to generate the estimated effect sizes for the predicted association between hospital activity and activity in the community

We find that appointments in General Practice and A&E attendances are substitutes since an one-unit change in General Practice appointments is associated with a − 0.38 [95% CI − 0.74, − 0.01] unit change in A&E attendances. We find no relationship between appointments in General Practice and non-elective inpatient admissions [− 0.02; 95% CI − 0.13, 0.08].

We find that appointments in General Practice and hospital admissions and outpatient visits have a complementary relationship where a one-unit change in appointments in General Practice is associated with an additional 0.09 [95% CI − 0.01, 0.18] unit change in hospital admission and 0.11 [95% CI − 0.02, 0.23] unit change in outpatient attendances. Full regression results are provided in Appendix Table A3.

### Net unit costs

Table 3 shows the net unit cost for community services is £34.16, which is lower than a unit cost of a community care contact of £64 [36]. We estimated that each additional community care contact lowers the expected cost of hospital services on the NHS by around £30; this is mainly driven by the expected reduction for inpatient hospital admission (£17). The net unit cost of an additional appointment in General Practice is £41. Due to the complementary nature of appointment in General practice and hospital services (hospital admission and first outpatient attendances), each additional appointment in General Practice is expected to increase the cost of hospital services to the NHS by around £11. However, because the estimated relationship between hospital services and appointments in General Practice is estimated with considerable uncertainty, the estimated net system cost has a wide 95% confidence range of between £8 and £74.

**Table 3:** Net, direct and indirect costs of services provided in the community

Services	Direct costs (£)	Indirect costs (£)				Net costs (£)
		A&E attendances	Non-elective hospital admissions	Inpatient admissions	First outpatient attendance	
Community care contacts	64	− 5.9 [− 8.1, − 3.7]	− 6.4 [− 11.3, − 0.5]	− 17.0 [− 28.0, − 5.6]	− 0.6 [− 1.1, − 0.1]	34.2 [15.5, 54.2]
Appointments in general practice	30	− 4.9 [− 9.6, − 0.1]	− 2.0 [− 10.4, 6.4]	16.9 [− 2.0, 35.5]	0.8 [− 0.2, 1.7]	40.9 [7.9, 73.6]

Estimated coefficients (Table 2) and mean activity levels (Table 1) were used to calculate the net unit cost using Eq. 9. Direct costs were obtained from NHS England and Improvement for hospital activity and community care [28] and average costs of appointment in General Practice [29]. Indirect costs are costs incurred by NHS for hospital services that are associated with activity in the community. All values displayed in the table are currencies of the Great British Pound Sterling. Therefore, a value of -5.9 for A&E attendance and community care contacts signifies that the indirect cost saving of a community care contact on A&E activity is £5.90

### Bias adjusted coefficients

For all of the relationships estimated to be substitutes, the effect of adjusting for a greater set of confounders would strengthen the substitution effect (Table 4). This suggests that the substitution effects shown by the analysis are the lower bounds. The two exceptions are the substitution effects for A&E and inpatient attendance with community care; however, the estimated coefficients' changes are small.

**Table 4:** Oster bias adjusted coefficients

Importance of unexplained variation	Index of relative GP appointments activity				Index of relative community care activity			
	Index of relative A&E attendances activity	Index of relative non-elective hospital admissions activity	Index of relative hospital admissions activity	Index of relative outpatient visits activity	Index of relative A&E attendances activity	Index of relative non-elective hospital admissions activity	Index of relative hospital admissions activity	Index of relative outpatient visits activity
0.1	− 0.394	− 0.034	0.067	0.084	− 0.161	− 0.029	− 0.030	− 0.029
0.2	− 0.412	− 0.043	0.048	0.063	− 0.161	− 0.030	− 0.029	− 0.032
0.3	− 0.431	− 0.053	0.029	0.042	− 0.160	− 0.030	− 0.028	− 0.035
0.4	− 0.449	− 0.062	0.011	0.021	− 0.159	− 0.031	− 0.028	− 0.038
0.5	− 0.468	− 0.071	− 0.008	0.000	− 0.159	− 0.032	− 0.027	− 0.040
0.6	− 0.486	− 0.080	− 0.027	− 0.021	− 0.158	− 0.032	− 0.026	− 0.043
0.7	− 0.505	− 0.089	− 0.046	− 0.041	− 0.157	− 0.033	− 0.025	− 0.046
0.8	− 0.523	− 0.099	− 0.065	− 0.062	− 0.156	− 0.034	− 0.025	− 0.049
0.9	− 0.542	− 0.108	− 0.083	− 0.083	− 0.156	− 0.034	− 0.024	− 0.052
1	− 0.560	− 0.117	− 0.102	− 0.104	− 0.155	− 0.035	− 0.023	− 0.054

This table contains coefficients from the main analysis (Table 2) that have been adjusted in line the Oster bias adjustment. Movements in coefficients and r-squared values are used to predict the direction and size of bias of not adjusting for all possible confounders. We calculated how our estimated coefficients will change when increasing the importance of the unexplained variation compared to explained variation from 0.1 (one tenth) to 1 (equal)

The estimated relationship between appointments in General Practice and total inpatient admissions and outpatient visits are not stable. As the importance of unexplained variation increases, we find that the complementary relationship becomes a substitute; however, the main findings are weak with statistical significance at the 5% level.

### Additional analyses

Table 5 shows results from four additional analyses focusing on rurality, same community and hospital provider, NHS providers and interpolating data. The complementary and substitute relationships between services provided in the community and hospital services are generally larger for hospital providers serving relatively rural populations than urban populations, except for A&E activities. When comparing the results from the main analysis with the results from using only providers that provide both hospital and community care, we find that the substitution effect is larger between care provided in the community and A&E activity. When imputing missing community provider data for months where providers did not submit data, estimates for GP appointments are relatively constant, but associations with community care and hospital services weaken. The results using NHS hospital providers only are consistent and similar to the main analysis.

**Table 5:** Additional analysis

	Main results	Rural providers only	Urban providers only	Same hospital and community provider	NHS Hospital Providers only	Interpolated community data	
						Main results	Same hospital and community provider
Index of relative A&E attendances activity							
Index of relative community contacts activity	− 0.162 [− 0.22,-0.10]	− 0.147 [− 0.23, − 0.07]	− 0.18 [− 0.26, − 0.10]	− 0.245 [− 0.34, − 0.15]	− 0.148 [− 0.21, − 0.08]	− 0.0239 [− 0.04, − 0.01]	− 0.0547 [− 0.08, − 0.03]
Index of relative GP appointments activity	− 0.375 [− 0.74, − 0.01]	− 0.286 [− 0.82, 0.24]	− 0.612 [− 1.45, 0.23]	− 0.491 [− 1.39, 0.40]	− 0.362 [− 0.73, 0.01]	− 0.37 [− 0.74, − 0.002]	− 0.152 [− 0.54, 0.24]
Provider months	3557	1560	1997	557	3414	3557	1232
Index of relative non-elective hospital admissions activity							
Index of relative community contacts activity	− 0.028 [− 0.05, − 0.002]	− 0.088 [− 0.14, − 0.04]	− 0.007 [− 0.04, 0.03]	− 0.047 [− 0.10, 0.01]	− 0.029 [− 0.06, − 0.002]	− 0.006 [− 0.01, 0.002]	− 0.015 [− 0.05, 0.02]
Index of relative GP appointments activity	− 0.025 [− 0.13, 0.08]	− 0.138 [− 0.30, 0.02]	0.017 [− 0.11, 0.15]	0.409 [0.01, 0.81]	− 0.026 [− 0.13, 0.08]	− 0.032 [− 0.14, 0.07]	0.131 [− 0.18, 0.44]
Provider months	3899	1738	2161	593	3844	3899	1244
Index of relative hospital admissions activity							
Index of relative community contacts activity	− 0.03 [− 0.05, − 0.01]	− 0.061 [− 0.09, − 0.03]	− 0.016 [− 0.04, 0.01]	− 0.01 [− 0.04, 0.02]	− 0.03 [− 0.05, − 0.01]	− 0.002 [− 0.01, 0.005]	0.002 [− 0.03, 0.03]
Index of relative GP appointments activity	0.086 [− 0.01, 0.18]	0.064 [− 0.09, 0.22]	0.079 [− 0.02, 0.18]	0.226 [0.004, 0.45]	0.084 [− 0.01, 0.18]	0.084 [− 0.01, 0.18]	0.301 [0.03, 0.58]
Provider months	4408	1942	2466	683	3958	4406	1346
Index of relative outpatient visits activity							
Index of relative community contacts activity	− 0.026 [− 0.05, − 0.005]	− 0.049 [− 0.08, − 0.02]	− 0.011 [− 0.04, 0.02]	0.037 [− 0.01, 0.09]	− 0.026 [− 0.05, − 0.004]	− 0.005 [− 0.02, 0.005]	0.014 [− 0.02, 0.05]
Index of relative GP appointments activity	0.105 [− 0.02, 0.23]	0.239 [0.06, 0.42]	0.022 [− 0.13, 0.17]	0.225 [− 0.02, 0.47]	0.091 [− 0.03, 0.21]	0.104 [− 0.02, 0.23]	− 0.077 [− 0.44, 0.28]
Provider months	4486	2000	2486	646	3983	4484	1372

The results from linear regression models. Models also include provider fixed effects, monthly time effects, proportion of population aged 0–14, 65 and over, proportion mal and index for relative CCG size. Indexes of relative activity are calculated by dividing monthly volume by the average volume reported by the provider over all months. All regressions are estimated with robust standard errors

Table 6 shows the sensitivity analysis results on exploring the use of different distance-size powers on the distance decay function ${\text{DW}}_{{{\text{ph}}}}$. We find that the results are largely robust to changing the distance function. Community service contacts are all estimated to have a substitution effect with all hospital services, the substitution being strongest between community contacts and A&E attendances activity and weakest between community activity and outpatient visits. Generally, increases in the decay function's power lead to reductions in the association between community and hospital services.

**Table 6:** Sensitivity analysis on exploring different powers on the distance decay function for the Index of relative community contacts activity

	Index of relative A&E attendances activity	Index of relative non-elective hospital admissions activity	Index of relative hospital admissions activity	Index of relative outpatient visits activity
Coefficient [95% confidence interval]				
Distance power 1	− 0.401 [− 0.536, − 0.265]	− 0.068 [− 0.130, − 0.005]	− 0.051 [− 0.081, − 0.021]	− 0.059 [− 0.092, − 0.027]
Distance power 1.5	− 0.232 [− 0.312, − 0.152]	− 0.039 [− 0.075, − 0.003]	− 0.0395 [− 0.062, − 0.017]	− 0.044 [− 0.069, − 0.018]
Distance power 2*	− 0.162 [− 0.222, − 0.102]	− 0.028 [− 0.055, − 0.002]	− 0.030 [− 0.049, − 0.011]	− 0.026 [− 0.048, − 0.005]
Distance power 2.5	− 0.126 [− 0.175, − 0.078]	− 0.025 [− 0.049, − 0.001]	− 0.027 [− 0.045, − 0.009]	− 0.016 [− 0.036, 0.003]
Distance power 3	− 0.103 [− 0.145, − 0.061]	− 0.024 [− 0.047, − 0.001]	− 0.025 [− 0.043, − 0.008]	− 0.011 [− 0.029, 0.007]
Distance power 5	− 0.062 [− 0.099, − 0.025]	− 0.021 [− 0.045,0.003]	− 0.024 [− 0.040, − 0.007]	− 0.001 [− 0.018, 0.016]

The results from linear regression models. Models also include provider fixed effects, monthly time effects, proportion of population aged 0–14, 65 and over, proportion male, income and relative index of CCG population. Indexes of relative activity are calculated by dividing monthly volume by the average volume reported by the provider over all months

*Distance power of 2 is used for the main analysis

The results from models using logged activity measures are included in Appendix Table A4. We find that community care contact and hospital services are substitutes, although only the effect of A&E activity is statistically significant at the 95% level. Similar to the main results, GP appointments are substitutes for emergency hospital activities and complements for elective hospital admissions. GP appointments are also complements for first outpatient visits, a relationship that is statistically significant at the 95% level.

## Conclusion

Utilising new national data, we examined the frequently suggested notion that community activity acts as a complement or a substitute for hospital services. We find that there is a weak substitution effect between community care contacts and all hospital services, with an additional community care contact (direct cost of £64) associated with a decrease in hospital costs to the NHS of £30 [95% CI £10, £48]. This weak substitution means that each additional community care contact is expected to increase net system costs by £34 [95% CI £6, £54]. We find the substitution effects are weak in magnitude, with 28 [95% CI 21, 45] community care contacts associated with a reduction of one A&E attendance and 517 [95% CI 291, 7265] community care contacts associated with a reduction of one non-elective admission. However, we are unable to estimate whether these initial increase in costs are due to meeting unmet needs or result in lower long-term health costs by potentially identifying the health needs of the population within the community. All our findings are robust to changes in the distance decay function when attributing community service to hospitals.

Appointments in General Practice and A&E attendances (34 [95% CI 17, 1283] GP appointments are associated with a reduction of one A&E attendance) and non-elective hospital admissions (1655 [95% CI − 1995, 70145] GP appointments are associated with a reduction of one non-elective admission) have a substitution relationship. Appointments in General Practice and total hospital admission and first outpatient visits are weak complements (the largest relationship being 241 [95% CI − 2719, 256] GP appointments being associated with an increase in one hospital admission), but this finding is not robust to the possibility of unobserved confounders. We estimate that overall net system costs per appointment in General Practice is £41 [95% CI £8. £74] (direct cost of £30) because an additional appointment in General Practice is associated with an increase in hospital costs to the NHS of £11 [95% CI − £22, £44].

The substitutability between community care and hospital services is stronger for providers that serve higher levels of rural populations compared to urban populations except for A&E. This may be due to the access to services available to the population and therefore the relative importance of such health care services in the community for the population in rural settings. With A&E services not being determined by a gatekeeper, the lack of availability of A&E in rural settings will render the service a weaker substitute.

These findings imply that increases in community services could relieve pressures on the volume of care delivery for hospitals. Suppose the assumption that increases in community care delivery could identify greater unmet need levels are correct. In that case, these findings suggest that the increases in community care provision are associated with lowering hospital activity despite providing care to patients with newly identified needs. The substitution between services provided in the community and A&E services are shown to have the largest substitution effects indicating that access to care services is more of an issue for people attending A&E services.

We would expect appointments in General Practice and hospital admissions and first outpatient attendances in England to be complementary. The GP being a gatekeeper for access to secondary care means that most outpatient and hospital admissions would have to be first initiated through a GP referral. So higher appointments could lead to higher levels of hospital admissions and outpatient visits. However, this result is not consistent with the large body of literature [4] where 11 out of 14 studies found a substitution between outpatient visits and specialist GP care, where 10 of the 11 studies were carried out in the UK. However, studies in the systematic review [4] assessed the impact of GPs acting as specialists and therefore as direct substitutes. In contrast, we consider total volumes of GP activity which may stimulate more demand for hospital care. On the other hand, we find that our results are weak complements where the inclusion of a greater set of confounders may likely diminish our findings of complementarity.

Our finding that community care and first outpatient appointment are substitutes is consistent with the literature on integrated care within the UK. However, finding of a substitution effect between community care and hospital admissions adds to a literature base in which there is no current consensus [19]. A possible explanation for this is that health care services delivered in the community may not be directly substitutable for all types of care performed within the hospital. Non-surgical first outpatient visits may be more responsive to the supply of care in the community as management of conditions may not require specialist attention. In comparison, elective surgery for a hip replacement is care that is non-substitutable. Urwin et al. [38] found that informal care can substitute for home help in the community, but not GP visits or inpatient hospital activity where the latter two services require treatment from more trained individuals.

### Strengths

To our knowledge, this is the first published analysis to use population-wide activity levels from the care provided in the community and hospital activity. The availability of two new datasets means that relationships between care delivered in the community and care delivered from hospitals can now be empirically tested. This is the first study to take population-wide community services data and GP appointments data to look at the association of care provided in the community and hospital care.

Furthermore, adopting distance-size weights allows for datasets available in different aggregation units to be linked. We compared GP attendances after applying historical and distance-size weighting to test the distance-size weight's performance; we found that the two weighting methods resulted in correlations of 0.99 for A&E attendances, hospital admission and outpatient visits when the distance decay function was set to the power of 2. We also conduct sensitivity analysis to explore whether the main results change based on the increasing and decreasing the distance decay function, which changes the importance of the proximity between hospital and community services providers. Ideally, a population-wide individual-level dataset containing all healthcare services activity would be used for such analysis. However, this dataset does not currently exist in England.

### Limitations

Counts of community care contacts from the CSDS are not stratified by the type of care contact; we are unable to identify which services are provided from which provider. This means that we cannot refine the distance-size models based on the type of service used. Therefore, we are unable to perform analysis on subsets of patients where we would expect changes in community care provision to more directly affect the use of hospital services. Furthermore, online statistics report the number of care contacts that are not attended; this information is held on individual patient records, and as such, not available.

Data on appointments within General Practice captures only planned appointments and do not include appointments which are emergencies or appointments which were arrange outside of core practice opening hours [39]. This means that not all appointments within General Practice are captured in this study. The data on the appointments within General Practice for all planned, unplanned and outside of core opening hours was not available for all GP Practices in England during this study.

Using monthly aggregated hospital activity data means that the data does not allow for the stratification by types of hospital services. We would expect types of care from A&E, outpatients and hospital admission to be unavoidable. However, outcomes such as avoidable admissions [15] would be more sensitive to changes in care delivered in the community. Furthermore, we have data only on first outpatient visits; we have not tested the relationship between care in the community and an overall number of outpatients visits. We are also limited within our analysis regarding the severity of each of the hospital services being utilised. The data are not available on the volumes of activity for specific services or population groups that may have a stronger relationship with services provided in the community, such as wound care for older age groups. This should be a priority for future research when these data become available.

Owing to the aggregated nature of available data, we do not have data on service users' location. Instead, we use postcodes of service providers. This is not ideal for providers with many sites that could be widely dispersed over a large geographical area. Using postcodes for community service providers, although not ideal, can be used as the location of service users as community service providers have geographical boundaries for where they provide care. However, as geographical boundaries for community care from each care provider are diverse and likely to be changeable over time due to short commissioner–provider contracts [32], using geographical footprints may still not be informative when linking across providers of different units.

Our models do not include any population-level health characteristics. The absence of these health variables results in a positive bias towards the estimated relationship between hospital and community services. One reason why population health variables are not included is that these variables will need to be time-varying. Owing to using fixed-effects regressions, we do control for the average level of health, but we do not account for changes in health over time. We estimate coefficient stability to test how the bias would affect coefficients and show that substitution effects estimated are assumed to be the lower bound. However, we are not as confident with the complementary findings as controlling for unobserved characteristics may result in substitutability. One assumption when using coefficient stability by Oster (2019) is that the direction of bias when including known confounders addresses the same direction of bias for the unknown confounders.

### Policy implications

Expansion of care in the community may not result in a significant immediate fall in hospital service use. Despite estimating a substitute effect between hospital services and community care services, the estimated net unit cost of each additional unit of community activity results in higher overall costs to the healthcare system, as cost-saving within secondary care are not outweighed by the cost of care delivered in the community. There may be long term or lagged benefits, including better population health in the long term, but, in the short term, increasing care delivered in the community will not lead to reductions in health system expenditure.

With a fixed level of hospital beds, increasing health care services delivered in the community may not reduce overall hospital bed utilisation, as demand for hospital services outweighs the supply indicated by waiting times, which can be seen as a form of rationing within the English NHS. However, freed up hospital resources through an expansion of community care activity enables other individuals in need of health care to use hospital services, which may result in a reduction in competing demands for the same hospital bed. This could lead to a potential spill over effect of reduction in waiting times for other patients and may be observed in reduction of symptom duration and benefit of surgery [40].

### Areas of further research

This study is the first to use population-wide data on health care activity delivered in the community and healthcare activity delivered in hospitals. Future avenues of work could improve this study using patient-level data to analyse the relationship between community services activity and hospital services activity. Using individual-level data would allow conducting analysis on both the intensive and the extensive margin for services users of community care; this then could provide a more in-depth and meaningful extension to the findings in this study.

This study did not take into account for the quality of care provided in the community and how it might impact hospital service utilisation. Studies on primary care quality show it has an effect onto secondary care utilisation [14] [15]. This suggests that the relationship between care provided in the community and hospital utilisation may differ by different care quality levels.

## Supplementary Information

Below is the link to the electronic supplementary material.

Supplementary file1 (DOCX 277 kb)

Supplementary file2 (DOCX 31 kb)
