# Supplementary material for: Complements or substitutes? Associations between volumes of care provided in the community and hospitals
Source: Eur J Health Econ. 2021 Jun 17;22(8):1167–81. doi: 10.1007/s10198-021-01329-6 (PMC8526459; doi:10.1007/s10198-021-01329-6)
Supplement: Supplementary file 1 — Supplementary file1 (DOCX 277 kb) [file 10198_2021_1329_MOESM1_ESM.docx]

**Figure A1: Monthly variation in volumes of hospital activity**

Left panels show national totals. Right panels show mean, and 5th and 95th percentile, values of provider level relative activity indices.

Figure A2: Monthly variation in volumes of services provided in community

Left panels show national totals. Right panels show mean, and 5th and 95th percentile, values of provider level relative activity indices.

Figure A3: Monthly variation in relative community activity levels attached to hospital providers

GP appointments attached to hospitals using weights obtained from micro-data (left panel). Community care contacts attached to hospitals using weights based on distances and relative provider sizes (right panel).
