# Supplementary material for: Complements or substitutes? Associations between volumes of care provided in the community and hospitals
Source: Eur J Health Econ. 2021 Jun 17;22(8):1167–81. doi: 10.1007/s10198-021-01329-6 (PMC8526459; doi:10.1007/s10198-021-01329-6)
Supplement: Supplementary file 2 — Supplementary file2 (DOCX 31 kb) [file 10198_2021_1329_MOESM2_ESM.docx]

**Appendix**

Table A1: Table of definitions of all indices of relative activity

| ***Index of relative activity*** | ***Definition*** |
| --- | --- |
| *A&E attendances* | *Volume of A&E attendances for each hospital services provider for each month, divided by the provider’s mean monthly volume of A&E attendances. The index is centred on one, with values greater than one signifying activity greater than the average activity for that provider. E.g. a value of 1.075 signifies a value of 7.5% higher than the average.* |
| *Non-elective inpatient admissions* | *Volume of non-elective inpatient admissions for each hospital services provider for each month, divided by the provider’s mean volume of non-elective inpatient admissions. Values are centred on one, with values greater than one signifying activity greater than the average activity for that provider.* |
| *Inpatient admissions* | *Volume of inpatient admissions for each hospital services provider for each month, divided by the provider’s mean volume of inpatient admissions. Values are centred on one, with values greater than one signifying activity greater than the average activity for that provider.* |
| *Outpatient visits* | *Volume of outpatient visits for each hospital services provider for each month, divided by the provider’s mean volume of outpatient visit. Values are centred on one, with values greater than one signifying activity greater than the average activity for that provider.* |
| *Community care contacts* | *Volume of community care contacts for each community services provider for each month, divided by the provider’s mean volume of community care contacts. This index is then attributed to hospital services providers though using community provider size and distance weights as explained in 'combining data sets’ section of the main manuscript.* |
| *GP appointments* | *Volume of GP appointments in each Clinical Commissioning Group for each month, divided by the Clinical Commissioning Group’s mean volume of GP appointments. This index is then attributed to hospital services providers though using historical weights as explained in 'combining data sets’ section of the main manuscript.* |

*Volume of hospital and community activity*

Figure A1 shows total national volumes of hospital activity in each month and the distribution of the provider-level indices of relative activity. There are, on average, 1.7 million emergency department attendances, 1.25 million hospital admissions (0.55 Million of which were non-elective) and 1.6 million outpatient visits per month. There is substantial monthly variability in the volume of activity for all types of hospital services. The largest amount of variation is observed in outpatient activity. The lowest levels of emergency department activity and non-elective admissions are in February. The lowest level of outpatient visits is in December.

There is large variability in the relative activity index between hospitals for all four hospital activity series. The difference between the 5^th^ percentile and 95^th^ percentile of the relative activity index is smallest for non-elective hospital admissions.

Figure A2 shows monthly national totals for appointments in General Practice and community care contacts. On average, there are 25 million appointments in General Practice and 2 million community care contacts per month. Activity is higher in General Practice in the winter months compared to summer months, with the exception of December. There is a downward trend in volume of appointments between January and August. The volume of community service contacts increases throughout the year until December, which has the lowest level of community activity.

Figure A2 also shows the variation in relative activity levels across providers of services in the community. There is low variability in relative volumes of appointments in General Practice between the CCGs at the 95th percentile and 5th percentiles, suggesting that CCG activity changes over time in similar ways. The relative activity index for community service providers varies more substantially. This reflects, in part, the larger differences in the sizes of community care providers, as small changes in activity for smaller providers create larger changes in indexed performance.

Figure A3 shows how the relative indices of activity levels in community-based services vary once they are attached to hospitals. There is less variation at this level than at the community service provider level because the figures are weighted-averages of the community provider level series. Nonetheless, there is more variation in activity in community services compared to hospital services.

<Insert Figure A1>

<Insert Figure A2>

<Insert Figure A3>

Distance-size weights calculation

Table A2 shows a hypothetical example of how the distance-size weights are calculated and used. In this example, there are two community service providers, P1 and P2, three hospital service providers, H1, H2 and H3 and two time periods. All three hospital providers are equidistant from P1 and therefore receive the same distance-size weight, ${GW}_{ph}$. Community provider P2 is assigned weights based on distance as size of P2 is a constant for P2.

Table A2: Distance-size weight example

| $t$ | $p$ | $h$ | $c_{pt}$ | $d_{ph}$ | $\bar{c}_{p}$ | $\frac{c_{pt}}{\bar{c}_{p}}$ | ${SW}_{p}$ | ${DW}_{ph}$ | ${GW}_{ph}$ | ${GW}_{ph}^{*}$ | $\frac{C_{pt}}{\bar{C}_{p}}*{GW}_{ph}^{*}$ | ${com}_{ht}$ |
| --- | --- | --- | --- | --- | --- | --- | --- | --- | --- | --- | --- | --- |
| 1 | P1 | H1 | 30 | 1 | 32.5 | 0.92 | 0.59 | 0.5 | 0.30 | 0.78 | 0.72 | 0.96 |
| 1 | P2 | H1 | 25 | 2 | 22.5 | 1.11 | 0.41 | 0.2 | 0.08 | 0.22 | 0.24 |  |
| 2 | P1 | H1 | 35 | 1 | 32.5 | 1.08 | 0.59 | 0.5 | 0.30 | 0.78 | 0.84 | 1.04 |
| 2 | P2 | H1 | 20 | 2 | 22.5 | 0.89 | 0.41 | 0.2 | 0.08 | 0.22 | 0.19 |  |
| 1 | P1 | H2 | 30 | 1 | 32.5 | 0.92 | 0.59 | 0.5 | 0.30 | 0.88 | 0.81 | 0.95 |
| 1 | P2 | H2 | 25 | 3 | 22.5 | 1.11 | 0.41 | 0.1 | 0.04 | 0.12 | 0.14 |  |
| 2 | P1 | H2 | 35 | 1 | 32.5 | 1.08 | 0.59 | 0.5 | 0.30 | 0.88 | 0.95 | 1.05 |
| 2 | P2 | H2 | 20 | 3 | 22.5 | 0.89 | 0.41 | 0.1 | 0.04 | 0.12 | 0.11 |  |
| 1 | P1 | H3 | 30 | 1 | 32.5 | 0.92 | 0.59 | 0.5 | 0.30 | 0.92 | 0.85 | 0.94 |
| 1 | P2 | H3 | 25 | 4 | 22.5 | 1.11 | 0.41 | 0.06 | 0.02 | 0.08 | 0.08 |  |
| 2 | P1 | H3 | 35 | 1 | 32.5 | 1.08 | 0.59 | 0.5 | 0.30 | 0.92 | 0.996 | 1.06 |
| 2 | P2 | H3 | 20 | 4 | 22.5 | 0.89 | 0.41 | 0.06 | 0.02 | 0.08 | 0.07 |  |

The weights are not purely determined by distance between H and P, and the size of P in isolation. Notice that ${GW}_{ph}^{*}$ for community provider P1 are no longer the same for all hospital service providers. This is because ${GW}_{ph}^{*}$ accounts for the relative distance and size of all P for a given H.

Full regression results

Table A3: Full regression results

|  | **Index of relative A&E attendances activity** | **Index of relative non-elective hospital admissions activity** | **Index of relative hospital admissions activity** | **Index of relative outpatient visits activity** |
| --- | --- | --- | --- | --- |
| **Index of relative community contacts activity** | -0.162 | -0.0281 | -0.0303 | -0.0263 |
|  | [-0.22,-0.10] | [-0.05,-0.002] | [-0.05,-0.01] | [-0.05,-0.005] |
| **Index of relative GP appointments activity** | -0.375 | -0.0249 | 0.0858 | 0.105 |
|  | [-0.74,-0.01] | [-0.13,0.08] | [-0.01,0.18] | [-0.02,0.23] |
| **Proportion aged 0-14** | -0.233 | 0.51 | 0.48 | 0.492 |
|  | [-3.14,2.68] | [0.10,0.92] | [0.13,0.83] | [0.13,0.85] |
| **Proportion aged 65 and over** | -0.786 | 0.121 | 0.134 | 0.141 |
|  | [-2.72,1.15] | [0.01,0.23] | [0.04,0.23] | [0.03,0.25] |
| **Proportion male** | -5.346 | 0.477 | 0.391 | 0.376 |
|  | [-10.83,0.14] | [-0.14,1.10] | [-0.15,0.93] | [-0.28,1.03] |
| **Index of relative CCG population size** | -3.606 | -0.964 | -0.615 | -0.00361 |
|  | [-4.85,-2.36] | [-1.47,-0.46] | [-0.97,-0.26] | [-0.48,0.47] |
| **Month (base=Nov-17)** |  |  |  |  |
| *Dec-17* | -0.101 | -0.00955 | -0.0893 | -0.216 |
|  | [-0.20,0.001] | [-0.04,0.02] | [-0.11,-0.06] | [-0.25,-0.18] |
| *Jan-18* | 0.00164 | 0.0332 | -0.0159 | -0.042 |
|  | [-0.05,0.05] | [0.01,0.05] | [-0.03,0.003] | [-0.06,-0.02] |
| *Feb-18* | -0.145 | -0.0837 | -0.0984 | -0.15 |
|  | [-0.22,-0.07] | [-0.11,-0.06] | [-0.12,-0.08] | [-0.17,-0.12] |
| *Mar-18* | 0.00124 | 0.0147 | -0.0281 | -0.113 |
|  | [-0.06,0.06] | [-0.01,0.04] | [-0.05,-0.01] | [-0.14,-0.09] |
| *Apr-18* | -0.0618 | -0.0326 | -0.0661 | -0.118 |
|  | [-0.14,0.01] | [-0.06,-0.01] | [-0.09,-0.05] | [-0.14,-0.09] |
| *May-18* | 0.0455 | 0.0262 | -0.00117 | -0.0349 |
|  | [-0.01,0.10] | [0.004,0.05] | [-0.02,0.02] | [-0.06,-0.01] |
| *Jun-18* | -0.00437 | -0.00408 | -0.016 | -0.0483 |
|  | [-0.08,0.07] | [-0.03,0.03] | [-0.04,0.01] | [-0.08,-0.02] |
| *Jul-18* | 0.0739 | 0.0351 | 0.0153 | -0.0162 |
|  | [0.01,0.14] | [0.01,0.06] | [-0.01,0.04] | [-0.04,0.01] |
| *Aug-18* | -0.0407 | 0.00939 | -0.0118 | -0.0854 |
|  | [-0.12,0.04] | [-0.02,0.04] | [-0.03,0.01] | [-0.11,-0.06] |
| *Sep-18* | 0.215 | -0.00888 | -0.0431 | -0.086 |
|  | [-0.22,0.65] | [-0.04,0.02] | [-0.07,-0.02] | [-0.12,-0.06] |
| *Oct-18* | 0.126 | 0.0799 | 0.0522 | 0.0444 |
|  | [0.07,0.18] | [0.05,0.10] | [0.03,0.07] | [0.02,0.07] |
| *Nov-18* | 0.102 | 0.0757 | 0.0454 | 0.0178 |
|  | [0.05,0.15] | [0.05,0.10] | [0.02,0.07] | [-0.01,0.04] |
| *Dec-18* | -0.0204 | 0.0608 | -0.0451 | -0.175 |
|  | [-0.12,0.08] | [0.03,0.09] | [-0.07,-0.02] | [-0.21,-0.14] |
| *Jan-19* | 0.112 | 0.109 | 0.0566 | 0.00805 |
|  | [0.06,0.17] | [0.08,0.13] | [0.04,0.08] | [-0.02,0.03] |
| *Feb-19* | -0.0279 | -0.0109 | -0.0363 | -0.0937 |
|  | [-0.10,0.04] | [-0.04,0.01] | [-0.06,-0.02] | [-0.12,-0.07] |
| *Mar-19* | 0.114 | 0.0864 | 0.0446 | -0.034 |
|  | [0.05,0.18] | [0.06,0.11] | [0.02,0.07] | [-0.06,-0.01] |
| *Apr-19* | 0.0689 | 0.0601 | 0.00376 | -0.0604 |
|  | [-0.01,0.15] | [0.03,0.09] | [-0.02,0.03] | [-0.09,-0.03] |
| *May-19* | 0.121 | 0.0914 | 0.0485 | -0.0133 |
|  | [0.05,0.19] | [0.07,0.12] | [0.03,0.07] | [-0.04,0.01] |
| *Jun-19* | 0.0624 | 0.0372 | 0.00224 | -0.0501 |
|  | [-0.02,0.15] | [0.01,0.07] | [-0.02,0.03] | [-0.08,-0.02] |
| *Jul-19* | 0.167 | 0.1 | 0.0769 | 0.0582 |
|  | [0.11,0.23] | [0.07,0.13] | [0.06,0.10] | [0.03,0.09] |
| *Aug-19* | 0.0481 | 0.0396 | 0.00279 | -0.0968 |
|  | [-0.04,0.14] | [0.01,0.07] | [-0.02,0.03] | [-0.13,-0.06] |
| *Sep-19* | 0.11 | 0.0466 | 0.00592 | -0.0257 |
|  | [0.04,0.18] | [0.02,0.07] | [-0.02,0.03] | [-0.05,0.0003] |
| **Constant** | 7.993 | 1.645 | 1.268 | 0.692 |
|  | [3.93,12.06] | [1.01,2.28] | [0.78,1.75] | [0.09,1.29] |
| **Provider-Months** | 3557 | 3899 | 4408 | 4486 |
| **R-Squared** | 0.0646 | 0.214 | 0.339 | 0.421 |

Additional sensitivity analysis

Table A4: Log-log models for health services activity

|  | **Logged index of relative A&E attendances activity** | **Logged index of relative non-elective hospital admissions activity** | **Logged index of relative hospital admissions activity** | **Logged index of relative outpatient visits activity** |
| --- | --- | --- | --- | --- |
| **Logged index of relative community contacts activity** | -0.076 | -0.0133 | -0.0138 | -0.018 |
|  | [-0.12,-0.03] | [-0.03,0.01] | [-0.03,0.005] | [-0.03,-0.004] |
| **Logged index of relative GP appointments activity** | -0.23 | -0.0247 | 0.0973 | 0.125 |
|  | [-0.47,0.01] | [-0.13,0.08] | [-0.002,0.20] | [0.001,0.25] |

Notes: Results from log log regression models. Models also include provider fixed effects, monthly time effects. Indexes of relative activity are calculated by dividing monthly volume by the average volume reported by the provider over all months. All regressions are estimated with robust standard errors. Estimated effect sizes are the number of contacts or appointments in the community that would change the use of hospital service by one. Effect sizes use the coefficients and mean activity levels to generate the predicted association between hospital activity and activity in the community.
